# Safety, tolerability, and immunogenicity of inactivated poliovirus vaccine with or without *E.coli* double mutant heat-labile toxin (dmLT) adjuvant in healthy adults; a phase 1 randomized study

**DOI:** 10.1016/j.vaccine.2023.01.048

**Published:** 2023-03-03

**Authors:** Rahsan Erdem, Ilse De Coster, Kanchanamala Withanage, Laina D. Mercer, Arnaud Marchant, Martin Taton, Nathalie Cools, Eva Lion, Fred Cassels, Deborah Higgins, Karen Ivinson, Emily Locke, Kutub Mahmood, Peter F. Wright, Chris Gast, Jessica A. White, Margaret E. Ackerman, Jennifer L. Konopka-Anstadt, Bernardo A. Mainou, Pierre Van Damme

**Affiliations:** a PATH, Center for Vaccine Innovation and Access, Seattle, WA, USA; b Vaccine & Infectious Disease Institute, Centre for the Evaluation of Vaccination, University of Antwerp, Edegem, Belgium; c Institute for Medical Immunology, Université libre de Bruxelles, Brussels, Belgium; d Vaccine & Infectious Disease Institute, Laboratory of Experimental Hematology, University of Antwerp, Wilrijk, Belgium; e Dartmouth College, Lebanon, NH, USA; f Division of Viral Diseases, Centers for Disease Control and Prevention, Atlanta, GA, USA

**Keywords:** Poliovirus, vaccine, inactivated poliovirus vaccine (IPV), dmLT, intestinal immunity

## Abstract

**Background:**

Inactivated trivalent poliovirus vaccine (IPV) induces humoral immunity, which protects against paralytic poliomyelitis but does not induce sufficient mucosal immunity to block intestinal infection. We assessed the intestinal immunity in healthy adults in Belgium conferred by a co-formulation of IPV with the mucosal adjuvant double mutant Labile Toxin (dmLT) derived from *Escherichia coli*.

**Methods:**

Healthy fully IPV-vaccinated 18–45-year-olds were randomly allocated to three groups: on Day 1 two groups received one full dose of IPV (n = 30) or IPV + dmLT (n = 30) in a blinded manner, and the third received an open-label dose of bivalent live oral polio vaccine (bOPV types 1 and 3, n = 20). All groups received a challenge dose of bOPV on Day 29. Participants reported solicited and unsolicited adverse events (AE) using study diaries. Mucosal immune responses were measured by fecal neutralization and IgA on Days 29 and 43, with fecal shedding of challenge viruses measured for 28 days. Humoral responses were measured by serum neutralizing antibody (NAb).

**Results:**

Solicited and unsolicited AEs were mainly mild-to-moderate and transient in all groups, with no meaningful differences in rates between groups. Fecal shedding of challenge viruses in both IPV groups exceeded that of the bOPV group but was not different between IPV and IPV + dmLT groups. High serum NAb responses were observed in both IPV groups, alongside modest levels of fecal neutralization and IgA.

**Conclusions:**

Addition of dmLT to IPV administered intramuscularly neither affected humoral nor intestinal immunity nor decreased fecal virus shedding following bOPV challenge. The tolerability of the dose of dmLT used in this study may allow higher doses to be investigated for impact on mucosal immunity.

Registered on ClinicalTrials.gov - NCT04232943.

## 1 Introduction

Following the eradication of smallpox, the extensive use of vaccines has nearly achieved the global eradication of a second human infectious disease, paralytic poliomyelitis. Wild polioviruses (WPVs) type 2 and 3 have been officially declared eradicated globally [1], with WPV type 1 endemic only to Afghanistan and Pakistan [2]. Most cases of paralytic poliomyelitis are now caused by rare cases of vaccine-associated paralytic poliomyelitis (VAPP) or, more frequently, due to circulating vaccine-derived polioviruses (cVDPV) reacquiring neurovirulence during passage through the intestines of vaccinees and their contacts in under-immunized communities [3]. As most cVDPV cases are due to Sabin type 2 virus circulating in environments conducive to transmission, the Global Polio Eradication Initiative (GPEI) coordinated a global effort to cease routine use of live Sabin type 2 vaccine following global eradication of WPV2. This step in the eradication strategy involved replacement of Sabin trivalent live oral poliovirus vaccines (tOPV) with a combination immunization schedule of bivalent live vaccine (bOPV; types 1 and 3) and at least one dose of inactivated poliovirus vaccine (IPV). The removal of Sabin type 2 from routine use was completed globally in May 2016 [4]. However, following the tOPV to bOPV switch, population-level immunity to type 2 decreased, leaving communities susceptible to new cVDPV2 outbreaks, resulting from ongoing pre-cessation chains of transmission and outbreak response immunizations with monovalent OPV2 [6]. cVDPV2 outbreaks remain a major challenge to eradication with 1,081 and 682 cases of cVDPV2 confirmed from 24 and 22 countries in the recent 2020 and 2021 peaks, respectively. Several strategies are being used to address cVDPV2, including the recent introduction of novel OPV2 (nOPV2) under a WHO Emergency Use Listing for outbreak response.

Additionally, administration of IPV in routine immunization is critical for the successful replacement of tOPV with bOPV. Although IPV protects the recipient from symptomatic disease through humoral immunity, it does not stimulate the robust mucosal immunity necessary at intestinal sites to arrest shedding [5]. In the period since OPV2 cessation, however, a global IPV shortage has limited and delayed supplies in low- and middle-income countries [7].

One strategy to address supply shortages and limited intestinal immunity induced by IPV is the development of adjuvanted inactivated vaccines enabling use of fractional antigen quantities (dose sparing) while improving intestinal immunity [8]. Fractional dosing has been investigated in clinical studies using an established vaccine adjuvant, aluminum hydroxide [9], [10], but no mucosal activity was observed. More recently, double mutant Labile Toxin (dmLT), a protein toxoid derived from wild-type Enterotoxigenic *Escherichia coli* (ETEC) labile toxin (LT), has been shown to have mucosal adjuvant effects in preclinical [11], [12], [13] and early phase clinical studies [5], [14], [15]. This phase 1 clinical trial investigated the safety of dmLT-adjuvanted IPV (IPV + dmLT) in healthy adults, as well as the humoral and intestinal immune responses to a full dose of IPV with or without dmLT relative to bOPV vaccination, including the impact on fecal viral shedding following a bOPV challenge.

## 2 Methods

This was a single-center phase 1 randomized study to compare the safety, tolerability, and immunogenicity of a single dose of IPV with or without dmLT in healthy adults. The study was conducted at the Centre for the Evaluation of Vaccination, Vaccine and Infectious Disease Institute, University of Antwerp, Belgium following approval of the Antwerp University Hospital Ethics Committee. It was performed in accordance with the Declaration of Helsinki and the ICH GCP and guidelines of the Federal Agency for Medicines and Health Products (FAMHP), Belgium. The primary objectives were to evaluate and compare the safety of IPV + dmLT versus IPV alone and to compare the rate of fecal viral shedding throughout 28 days after a bOPV challenge dose at Day 29 post vaccination. The key secondary objectives were the evaluation and comparison of intestinal immune responses and the extent of fecal viral shedding following bOPV challenge.

Eligible participants were healthy 18–45-year-old males or females with a history of complete IPV vaccination (at least three doses of IPV) who were available for the duration of the study. Main exclusion criteria were receipt of OPV at any time or IPV vaccination within the previous 5 years, having routine contact with children incompletely vaccinated against polio, i.e., those under 6 months of age, or any known conditions that might interfere with immune responses. IPV or IPV + dmLT were administered intramuscularly on Day 1 to groups of 30 participants each, in a blinded manner. A positive control group (unblinded) was included, composed of 20 adults who received bOPV. A challenge dose of bOPV was given to all participants on Day 29. Eight participants per day (the maximum capacity of the study site) were randomized in a 3:3:2 ratio to one of the three treatment groups, IPV, IPV + dmLT, and bOPV, using a permuted-block design generated and maintained by the Statistical Data Coordinating Center (SDCC) at The Emmes Company, LLC (Emmes). Subsets of 10 participants per group, one per group per day, were randomly selected for assessment of antibody secreting cells (ASC) α_4_β_7_ integrin gut homing marker.

### 2.1 Vaccines

The licensed trivalent Salk IPV used was IMOVAX®-Polio (Sanofi Pasteur, France); each 0.5 mL dose contains 40 D-antigen units of type 1 (Mahoney strain), 8 DU type 2 (MEF-1 strain) and 32 DU type 3 (Saukett strain) polioviruses produced in VERO cells. The bOPV vaccine was Bivalent Polio Sabin™ One and Three produced by GSK (Rixensart, Belgium); each 0.1 mL oral dose contained not less than 10^6.0^ CCID_50_ of type 1 and 10^5.8^ CCID_50_ of type 3 polioviruses. The adjuvant dmLT (lot 001-08-16), also known as LT (R192G/L211A), was manufactured by IDT Biologika (Dessau-Rosslau, Germany). The IPV + dmLT formulation was prepared under aseptic conditions by an unblinded qualified research pharmacist at the clinical site. On the day of administration, a single vial of lyophilized dmLT was rehydrated with 0.5 mL of Sterile Water for Injection to produce a 1 mg/mL stock solution. Serial dilutions of dmLT were performed with pooled IMOVAX® Polio vaccine, by combining the contents of single-dose syringes (0.5 mL) in a sealed, sterile glass vial. Diluted dmLT was mixed with pooled IMOVAX® Polio vaccine in a quantity sufficient to vaccinate all scheduled participants on the day of preparation. The final IPV + dmLT formulation contained 0.5 µg of dmLT per 0.5 mL dose.

### 2.2 Endpoints

The primary safety endpoints were the frequencies and incidences of serious adverse events (SAEs) throughout the study, unsolicited adverse events (AE), especially those graded as severe during the 28 days following study vaccination and solicited reactogenicity (local and systemic reactions) during the 7 days following vaccination and challenge. The primary efficacy endpoint, the proportion of participants without detectable fecal shedding of bOPV vaccine viruses in the IPV + dmLT and IPV arms, 7 days after challenge, was chosen as a direct measure of the intestinal immunity conferred by vaccination.

Secondary endpoints included the proportions of participants with type-specific poliovirus fecal IgA and neutralizing responses 28 days after vaccination and 14 days after challenge; the serum neutralizing antibody (NAb) seroconversion rate and NAb levels 28 days after vaccination with IPV + dmLT or IPV; the area under the curve (AUC) of fecal shedding measured by CCID_50_ per gram of stool in the 28 days following challenge; and the proportions of participants developing type-specific poliovirus antibody-secreting cell (ASC) responses at any time point following both vaccination and challenge.

### 2.3 Safety

SAEs evaluated throughout the study were any events resulting in death or were life-threatening, required hospitalization, and/or resulted in a persistent incapacity that disrupted normal life. General health and clinical laboratory assessments—complete blood counts (CBC) with differential for white blood cell (WBC), hemoglobin, absolute neutrophil count (ANC), platelets, creatinine, albumin, total bilirubin, alanine transaminase (ALT), aspartate aminotransferase (AST), C-reactive protein (CRP), and antibodies against HBsAg, HIV and HCV—were performed during screening before vaccination, and on Day 8 post-vaccination for serum chemistry and hematology. Solicited local injection site reactions were pain, erythema/redness, swelling, induration and hyperpigmentation for the two IPV arms, and solicited systemic adverse events were chills, fatigue, headache, muscle aches/myalgia, joint ache/arthralgia, rash, nausea, vomiting, diarrhea, and fever defined as an oral temperature ≥ 38.0 °C for all participants. Unsolicited adverse events were reported from Day 1 to Day 57. Solicited and unsolicited AEs were graded for severity on a scale of 0 (normal), 1 (mild), 2 (moderate), and 3 (severe).

### 2.4 Biological samples

Blood and stool samples were temporarily stored at the Centre for the Evaluation of Vaccination (CEV) or in a central biorepository at the Laboratory of Experimental Hematology (LEH), Vaccine and Infectious Disease Institute, University of Antwerp after processing until they were shipped to the appropriate laboratories for analyses. Fresh whole blood was shipped to the Institute for Medical Immunology, Université Libre de Bruxelles (ULB, Brussels, Belgium) for determination of polio type-specific IgA/IgG ASC and ASC positive for the α_4_β_7_ integrin gut homing marker in a subset of samples [16]. Serum samples for poliovirus NAb and stool samples to assess for presence and quantity of shed virus were processed and temporarily stored frozen at the CEV for transportation on dry ice to the laboratory at the Centers for Disease Control and Prevention (CDC), Atlanta, GA, USA [17], [18]. Stool samples for fecal IgA and fecal NAb were processed and temporarily stored frozen at the CEV for transportation on dry ice to Dartmouth College (Geisel School of Medicine, Lebanon, NH, USA).

### 2.5 Viral shedding

Stool samples were obtained on Day 29 post-vaccination, before bOPV challenge, and then on Days 33, 36, 39, 43, 46, 50 and 57 (equivalent to Days 4, 7, 10, 14, 17, 21 and 28 post-bOPV challenge). Type-specific fecal viral shedding was assessed using reverse transcription polymerase chain reaction (RT-PCR) to detect viral RNA, and then total infectious virus, measured as 50% cell culture infective dose (CCID_50_), was titered in those positive for viral RNA by standardized methods at the CDC as previously described [19].

### 2.6 Immunogenicity endpoints

Titers of type-specific NAb on Days 1 and 29 post-vaccination (serum samples collected prior to vaccination and challenge, respectively) measured by standard micro-neutralization assay methods [17], [18] were expressed as the reciprocal of the highest serum dilution with no cytopathic effect (CPE). The seroconversion rate is defined as the proportion of participants demonstrating a minimum four-fold increase in type-specific poliovirus serum NAb titers between Days 1 and 29, or a Day 29 reciprocal neutralizing titer ≥8 if seronegative at baseline. Also calculated were geometric mean titers (GMT), geometric mean-fold rises (GMFR) between Days 1 and 29, and seropositivity (seroprotection) rates (proportions of each group with a titer ≥8) on Days 1 and 29.

Intestinal immunogenicity was measured as poliovirus fecal neutralization and fecal IgA in samples obtained at screening before vaccination, and then on Days 8, 29 (prior to challenge), 36, 43, 50, and 57 using standardized methods. Fecal neutralizing activity was measured by limiting dilution inhibition of luciferase-expressing wild-type-derived polio pseudoviruses and expressed as the titer needed to achieve 60% neutralization (titers >2 were considered detectable) [20]. Total and polio-type specific concentrations of fecal IgA were measured in a Luminex assay using monovalent IPV covalently conjugated to fluorescent coated beads [21]. The assay was developed using the Salk poliovirus strains from IPV vaccine, but for this study the assay was also run using the Sabin strains from IPV. Results are expressed as group proportions of participants who developed type-specific poliovirus fecal neutralization responses (minimum 4-fold increase from baseline) or fecal IgA and as GMTs and GMFR between baseline (Day 1, pre-vaccination) and post-baseline measurements on 29 days (pre-challenge) and 43 days (14 days after bOPV challenge).

Responses of type-specific poliovirus antibody-secreting cells (ASC) measured by a standard method [16] were defined as achieving ≥8 ASC/10^6^ PBMC at any time point following both study vaccination and bOPV challenge. Type-specific circulating IgG- and IgA-secreting α_4_β_7_ ASC homing markers were measured ex vivo by ELISPOT in randomly selected subsets of 10 samples per group. Briefly, after PBMC isolation, B cells were enriched by using the EasySep™ Human B Cell Enrichment Kit from Stemcell. After antibody staining and gating, a pattern of three populations of cells were sorted by flow cytometry and analyzed by ELISPOT: α_4_β_7_-, α_4_ β_7_ dim and α_4_ β_7_ bright, with the two latter populations considered positive. The GMT and frequency of type-specific poliovirus ASCs were calculated before and after study vaccination, as well as the GMFR between baseline and post-baseline measurements.

### 2.7 Statistics

With 30 participants per IPV group, this study had an 80% probability of detecting at least one AE that occurs at a rate of 5.3% or higher. With 27 evaluable participants per IPV arm, this study was designed to provide at least 96% power to detect ≥60% reduction in shedding rate 8 days post-challenge in the IPV + dmLT group assuming the shedding rate in the IPV alone group was at least 80%. All adverse events were summarized for the total vaccinated population, according to treatment received. All participant-level percentages were supplemented with two-sided 95% confidence intervals (CIs) computed via the Clopper-Pearson method.

The primary viral shedding endpoint was assessed in the per protocol population. The proportion of participants with stool positive for poliovirus was summarized by time point and group including corresponding 95% CIs. Proportions shedding in IPV groups were compared for each serotype and overall via one minus the relative risk and accompanied by a 95% CI computed using the Farrington and Manning method [22]. The type-specific time to cessation of shedding was analyzed by Kaplan-Meier methods, including right-censoring where appropriate. Quartiles of time to cessation of shedding and the shedding cessation rate at each post-challenge day were estimated along with corresponding 95% CIs, using the Greenwood method [23]. Cessation of viral shedding was defined as the day of the first PCR-negative stool for challenge virus after which the next two consecutive stool samples were also PCR-negative. Additionally, viral shedding (log_10_ CCID_50_/g, not type-specific) was summarized descriptively as a continuous variable with LLOQ (2.75 log_10_) and ULOQ (8.25 log_10_) used as the observed value whenever these limits were met and 0 for PCR-negative samples. A viral shedding index estimate was calculated using the arithmetic mean of the log_10_ CCID_50_/g samples collected on Days 36, 43, 50, and 57 and supplemented with the difference in medians (IPV + dmLT minus IPV alone) with corresponding two-sided 95% CIs computed using the percentile bootstrap method. The ratio of the shedding index was also calculated as the difference on the log scale, with accompanying 90% CI computed using the same bootstrap method, then back-transformed using the antilog. Here, the 90% CI is used to enable a one-sided level 0.05 non-inferiority test.

Immunogenicity assessments conducted in the per protocol population were summarized descriptively as GMTs, GMFRs, and seroresponse or seroconversion rates and compared between groups using baseline-adjusted GMT ratios. Geometric means were analyzed on the log scale, adjusted for baseline measures, and using survival regression analysis to accommodate censoring at LLOQ or ULOQ with antilog transformations of model-based estimates and corresponding 95% CIs.

## 3 Results

**Fig. 1:**
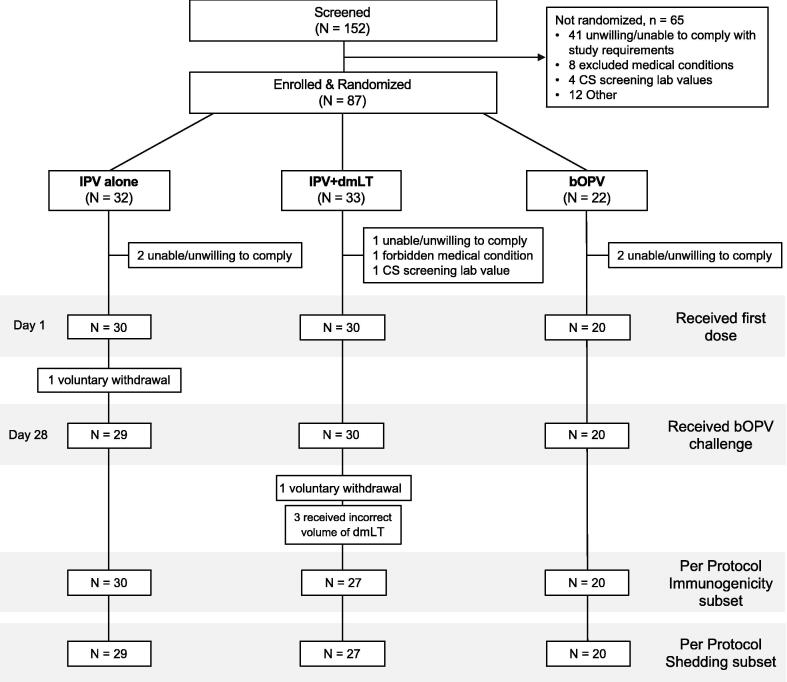
Study flow chart.

This study was initiated on January 22, 2020, but enrolment was halted on March 16, 2020 due to the COVID-19 pandemic and specific COVID-19 prevention measures instituted in Belgium at that time; the study resumed on July 27, 2020, with completion on February 1, 2021. A total of 152 volunteers were enrolled, of whom 87 were randomized to one of three groups to receive one dose of either IPV alone (n = 32), IPV + dmLT (n = 33) or bOPV (n = 22). As shown in Fig. 1**, 80** participants received a study vaccine; 60 received IPV with or without dmLT and 20 received bOPV. The numbers of participants eligible for the per protocol immunogenicity and shedding analyses were 77 (96%) and 76 (95%), respectively. Two participants voluntarily withdrew from the study for reasons unassociated with the study, with three excluded after dose verification indicated a reduced dose of dmLT had been administered (Fig. 1). The demographics of participants who received study vaccines were comparable across the three groups (Table 1) .

**Table 1:** Demographics of the total vaccinated study population.

	IPV (N = 30)	IPV + dmLT (N = 30)	bOPV (N = 20)
**Sex, n (%)**			
Male	18 (60.0)	17 (56.7)	13 (65.0)
Female	12 (40.0)	13 (43.3)	7 (35.0)

**Age, years**			
Mean (SD)	18.9 (1.61)	18.8 (1.35)	20.1 (4.18)
Minimum, maximum	18–27	18–25	18–33

**Ethnicity, n (%)**			
Hispanic or Latino	–	–	1 (5.0)
Not Hispanic or Latino	30 (100)	29 (96.7)	19 (95.0)
Unknown	–	1 (3.3)	–

**Race n, (%)**			
Black or African American	2 (6.7)	–	1 (5.0)
Native Hawaiian or Other Pacific Islander	–	–	1 (5.0)
White	28 (93.3)	30 (100)	18 (90.0)

**BMI, (kg/m^2^)**			
Mean (SD)	22.93 (3.71)	22.33 (3.10)	22.57 (2.97)
Minimum, maximum	17.6–34.0	17.0–27.6	18.5–27.6

**Table 2:** Unsolicited adverse events in the total vaccinated study population up to Day 29.

	IPV (N = 30)	IPV + dmLT (N = 30)	bOPV (N = 20)
All adverse events, n (%) *e* *			
Any AE	20 (67) *31*	18 (60) *46*	11 (55) *30*
Any severe AE	3 (10) *3*	4 (13) *4*	0
Any serious AE	0	0	0
Any AE leading to withdrawal	0	0	0

All adverse events within 28 days of vaccination, n (%) *			
Any	14 (47) *19*	15 (50) *24*	10 (50) *20*
Severe	2 (7) *2*	2 (7) *2*	0
Serious AE	0	0	0

All related adverse events within 28 days of vaccination, n (%) *			
Any	5 (17) *5*	4 (13) *6*	5 (25) *6*
Severe	0	1 (3) *1*	0
Serious AE	0	0	0

* n = number of participants reporting an AE; e = number of events			

### 3.1 Safety and reactogenicity

**Fig. 2:**
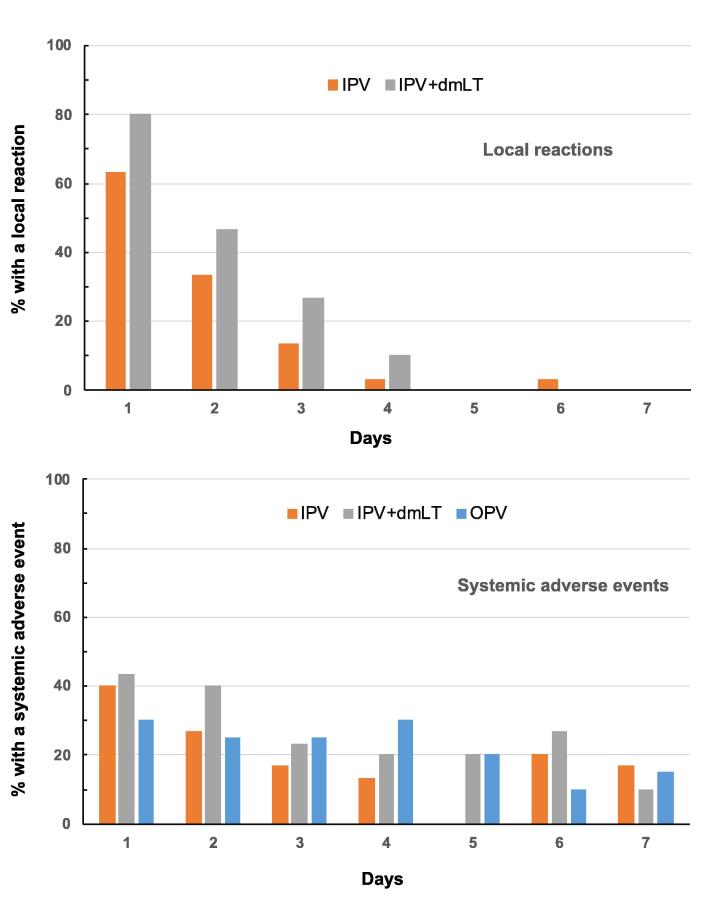
Frequencies of solicited local reactions and systemic adverse events in the study groups for 7 days after vaccination on Day 1.

Overall, study vaccinations were well tolerated with acceptable reactogenicity; there were no deaths, serious AEs or study withdrawals due to adverse events. Two reported immediate reactions within 30 min of vaccination were mild cases of headache in the IPV group and nausea in the IPV + dmLT group. On the day of vaccination, 19 (63%) of 30 IPV recipients and 24 (80%) of 30 IPV + dmLT recipients reported a local reaction, all graded as mild or moderate in severity. The majority of these reactions consisted of mild pain at the injection site with only two cases of induration (one in each group), a single case of swelling (IPV group) and single cases of erythema and hyperpigmentation (both in the IPV + dmLT group). Reports of local reactions declined at similar rates in both groups over the subsequent three days (Fig. 2).

Frequencies of solicited systemic AEs were comparable in all three groups, reported by 40%, 43% and 30% of IPV, IPV + dmLT and bOPV groups on Day 1, respectively (Fig. 2). Systemic AEs graded as moderate or severe were significantly more frequent on Day 1 (p = 0.029) in the IPV + dmLT group (6 events in 30 participants, 20%) than IPV (1 event in 30 participants, 3.3%) or bOPV (0 events). The most frequent systemic AEs were fatigue and headache, both reported by 11 (37%) of 30 IPV, 13 (43%) of 30 IPV + dmLT and 9 (45%) of 20 bOPV recipients. Rates of systemic AEs declined more gradually than local reactions and participants in all three groups continued to report them through Day 7 with no significant differences between study groups (Fig. 2), but all had resolved spontaneously by Day 15.

Unsolicited AEs up to Day 28 were reported by 20 (67%) of the 30 IPV recipients, compared with 18 (60%) of the 30 IPV + dmLT recipients and 11 (55%) of 20 who received bOPV. Unsolicited AEs were mainly mild or moderate in severity; although there were 3 and 4 events described as severe after IPV and IPV + dmLT, respectively; only one of these was considered to be related to vaccination – a case of severe transient elevated aspartate aminotransferase (AST) in an IPV + dmLT vaccinee which spontaneously resolved 10 days after first being observed. There were no other clinically significant changes from baseline or differences between treatment groups in laboratory values, vital signs, or physical examinations (see Table 2).

### 3.2 Stool viral shedding

**Fig. 3:**
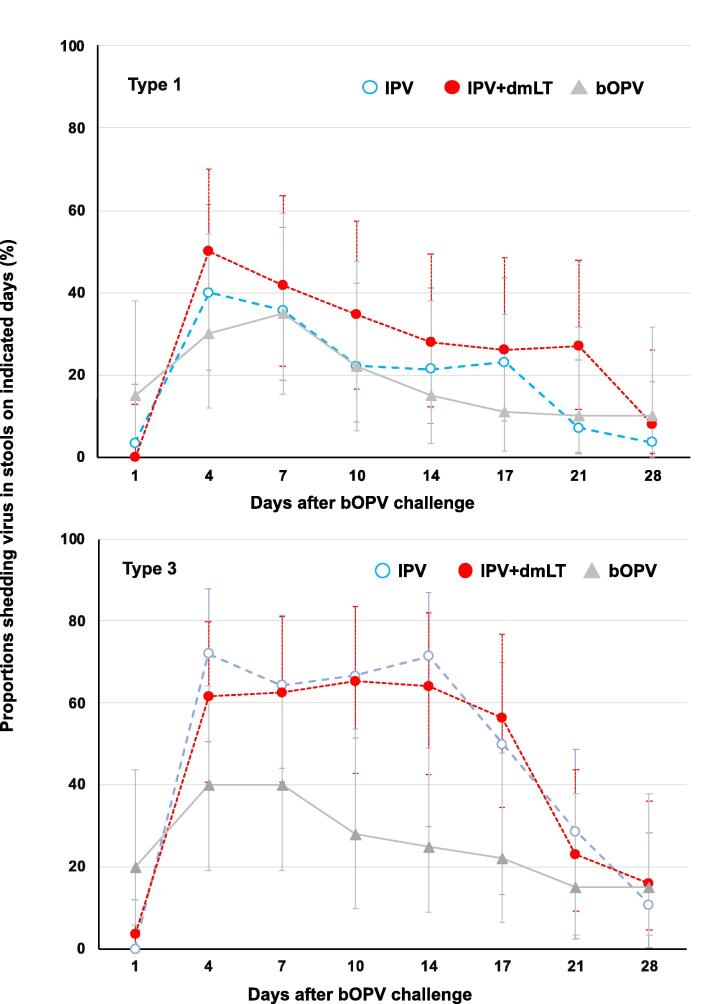
Shedding of poliovirus types 1 and 3 over the 28 days after challenge with bOPV in the three study groups. Shown as percentages of each group shedding with 95% CI bars.

Viral stool shedding peaked four days after bOPV challenge in all study groups regardless of virus type. For both types 1 and 3 the proportions of both IPV-treated groups who were shedding were similar through 28 days after bOPV challenge (Fig. 3). There was an observable trend to lower rates of shedding in the bOPV group, which was clearest for type 3, in which there was a lower rate in the bOPV group than the similar rates in IPV and IPV + dmLT groups. Shedding was indistinguishable and rare across all three groups by 28 days post-challenge. Median time to cessation of type 1 shedding was 6 days (95% CI: 5–9) for IPV, 7 days (95% CI: 5–14) for IPV + dmLT and 5 days (95% CI: 4–9) for bOPV groups. For type 3 the respective times were 9 days (95% CI: 4–18) for IPV, 19 days (95% CI: 10–27) for IPV + dmLT and 5 days (95% CI: 4–11) for bOPV.

At the predefined time-point of Day 36, 7 days after challenge, the relative risk (RR) for type-specific viral shedding (IPV + dmLT/IPV) was 1.17 (CI: 0.56–2.46) for type 1 and 0.97 (CI: 0.56–2.46) for type 3. Percentage reductions were −0.17 (CI: −1.464–0.440) and 0.03 (CI: −0.510–0.394) for poliovirus type 1 and 3, respectively. Confidence intervals for the RR contained 1.0 for treatment with IPV + dmLT compared with IPV alone and estimated risk reduction in shedding of any virus type was modest (<20%), suggesting no significant difference in viral shedding for either poliovirus type with the addition of dmLT.

### 3.3 Intestinal immunity

**Fig. 4:**
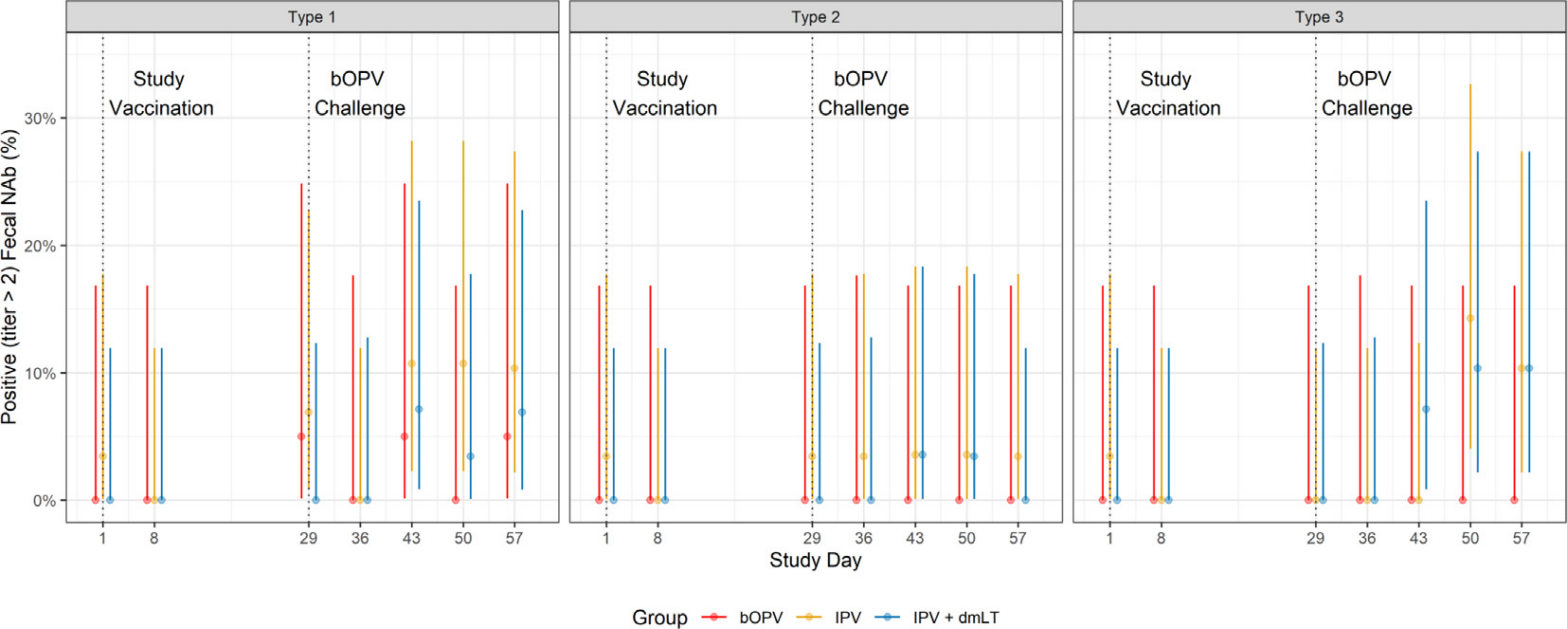
Presence of fecal type-specific poliovirus neutralization activity in the three study groups. Shown as percentages of each group with detected activity with 95% CI bars.

All positive fecal neutralization responses occurred after bOPV challenge except for one response in the IPV group before bOPV challenge (Fig. 4). Positive fecal neutralization responses to type 1 were detected in no more than two participants at any timepoint in IPV or IPV + dmLT groups, and there were no positive type 1 responses in any bOPV participant at any time up to Day 57. Type 3 responses were observed in no more than three participants at each timepoint in the IPV group, in no >2 participants in the IPV + dmLT group, and in only 1 participant in the bOPV group.

As with the fecal neutralizing responses, only a small proportion of participants demonstrated any measurable changes in fecal IgA over time. Generally, higher levels of fecal IgA were observed when measured using the Sabin strains which also resulted in more variable results than the Salk strains. However, fecal IgA levels using the Salk strains were higher in the IPV group compared with the IPV + dmLT group, particularly for serotypes 1 and 2, and on Day 29.

No meaningful differences were observed in IgG (Table 3) or IgA (Table 4) ASC cells between treatment groups. Large proportions of the IPV groups demonstrated IgG ASC against type 1 at Day 8 after vaccination, 90.0% and 74.1% in IPV and IPV + dmLT groups, respectively, with lower proportions against type 2 (56.7% and 51.9%) and type 3 (40.0% and 40.7%). Proportions with ASC for all three types were lower in the bOPV group (Table 3). IgG ASC were undetectable at Day 29 in all groups, and increases were much lower in all groups after bOPV challenge. The same profile of responses was observed for IgA ASC but with much lower proportions with detectable responses at Days 8 and 29, with the highest responses being observed for type 1 and within the bOPV group (Table 4).

**Table 3:** Proportions with type-specific circulating IgG antibody-secreting cells (ASC) by timepoint in each per protocol study group.

	IPV group		IPV + dmLT group		bOPV group	
Time	n/N %	(95% CI)^a^	n/N %	(95% CI)^a^	n/N %	(95% CI)^a^
**Poliovirus type 1**						
**Baseline**	0/30 **0**	(0.0–11.6)	0/27 **0**	(0.0–12.8)	0/20 **0**	(0.0–16.8)
**Day 8**	27/30 **90.0**	(73.5–97.9)	20/27 **74.1**	(53.7–88.9)	10/20 **50.0**	(27.2–72.8)
**Day 29**	0/29 **0**	(0.0–11.9)	0/26 **0**	(0.0–13.2)	0/20 **0**	(0.0–16.8)
**Day 36**	8/29 **27.6**	(12.7–47.2)	6/26 **23.1**	(9.0–43.7)	2/20 **10.0**	(1.2–31.7)

**Poliovirus type 2**						
**Baseline**	0/30 **0**	(0.0–11.6)	0/27 **0**	(0.0–12.8)	0/20 **0**	(0.0–16.8)
**Day 8**	17/30 **56.7**	(37.4–74.5)	14/27 **51.9**	(32.0–71.3)	2/20 **10.0**	(1.2–31.7)
**Day 29**	0/29 **0**	(0.0–11.9)	0/26 **0**	(0.0–13.2)	0/20 **0**	(0.0–16.8)
**Day 36**	1/29 **3.4**	(0.1–17.8)	1/26 **3.8**	(0.1–19.6)	0/20 **0**	(0.0–16.8)

**Poliovirus type 3**						
**Baseline**	0/30 **0**	(0.0–11.6)	0/27 **0**	(0.0–12.8)	0/20 **0**	(0.0–16.8)
**Day 8**	12/30 **40.0**	(22.7–59.4)	11/27 **40.7**	(22.4–61.2)	4/20 **20.0**	(5.7–43.7)
**Day 29**	0/29 **0**	(0.0–11.9)	0/26 **0**	(0.0–13.2)	0/20 **0**	(0.0–16.8)
**Day 36**	4/29 **13.8**	(3.9–31.7)	1/26 **3.87**	(0.1–19.6)	1/20 **5.0**	(0.1–24.9)

a. 95% CI = Confidence interval computed via Clopper-Pearson method. Positivity defined as a background-subtracted ASC count ≥ 8 cells per 10^6^ PBMC.

**Table 4:** Proportions with type-specific IgA antibody-secreting cells (ASC) by timepoint in each per protocol study group.

	IPV group		IPV + dmLT group		bOPV group	
Time	n/N %	(95% CI)^a^	n/N %	(95% CI)^a^	n/N %	(95% CI)^a^
**Poliovirus type 1**						
**Baseline**	0/30 **0**	(0.0–11.6)	0/27 **0**	(0.0–12.8)	0/20 **0**	(0.0–16.8)
**Day 8**	3/30 **10.0**	(2.1–26.5)	2/27 **7.4**	(0.9–24.3)	5/20 **25.0**	(8.7–49.1)
**Day 29**	0/29 **0**	(0.0–11.9)	0/26 **0**	(0.0–13.2)	0/20 **0**	(0.0–16.8)
**Day 36**	2/29 **6.9**	(0.9–22.8)	4/26 **15.4**	(4.4–34.6)	0/20 **0**	(0.0–16.8)

**Poliovirus type 2**						
**Baseline**	0/30 **0**	(0.0–11.6)	0/27 **0**	(0.0–12.8)	0/20 **0**	(0.0–16.8)
**Day 8**	2/30 **6.7**	(0.8–22.1)	1/27 **3.7**	(0.1–19.0)	2/20 **10.0**	(1.2–31.7)
**Day 29**	0/29 **0**	(0.0–11.9)	0/26 **0**	(0.0–13.2)	0/20 **0**	(0.0–16.8)
**Day 36**	1/29 **3.4**	(0.1–17.8)	2/26 **7.7**	(1.0–25.1)	0/20 **0**	(0.0–16.8)

**Poliovirus type 3**						
**Baseline**	0/30 **0**	(0.0–11.6)	0/27 **0**	(0.0–12.8)	0/20 **0**	(0.0–16.8)
**Day 8**	1/30 **3.3**	(0.1–17.2)	1/27 **3.7**	(0.1–19.0)	3/20 **15.0**	(3.2–37.9)
**Day 29**	0/29 **0**	(0.0–11.9)	0/26 **0**	(0.0–13.2)	0/20 **0**	(0.0–16.8)
**Day 36**	1/29 **3.4**	(0.1–17.8)	2/26 **7.7**	(1.0–25.1)	0/20 **0**	(0.0–16.8)

a. 95% CI = Confidence interval computed via Clopper-Pearson method. Positivity defined as a background-subtracted ASC count ≥ 8 cells per 10^6^ PBMC.						

Few α_4_β_7_ integrin gut homing ASCs were observed. Positive IgA ASC responses were more frequently observed to poliovirus type 1 (Table 5) than to types 2 or 3 (data not shown), and particularly in cells with high expression of the α_4_β_7_ marker; post-bOPV challenge 44.4% of participants in the IPV group, 42.9% in IPV + dmLT group, and 50% in the bOPV group had α_4_β_7_^high^ IgA ASCs to poliovirus type 1, but there were no participants with high levels of IgA homing ASCs to poliovirus type 2 or type 3.

**Table 5:** Proportions with type 1-specific circulating IgA and IgG-secreting α4β7 ASC Homing Marker 7 days after vaccination (Day 8) or bOPV-challenge (Day 29) in each per protocol study group striated according to expression (neg, dim and high).

		IPV group		IPV + dmLT group		bOPV group	
	Time	n/N %	(95% CI)^a^	n/N %	(95% CI)^a^	n/N %	(95% CI)^a^
**Homing IgG ASC**							
**Neg**	**Day 8**	7/10 **70%**	(35–93)	6/9 **67%**	(30–93)	0/10 **0%**	(0–31)
	**Day 29**	1/10 **10%**	(0–45)	0/9 **0%**	(0–34)	2/10 **20%**	(3–56)
**Dim**	**Day 8**	6/10 **60%**	(26–88)	7/9 **78%**	(40–97)	3/10 **30%**	(7–65)
	**Day 29**	2/10 **20%**	(3–56)	3/9 **33%**	(7–70)	2/10 **20%**	(3–56)
**High**	**Day 8**	5/10 **50%**	(19–81)	6/9 **67%**	(30–93)	4/9 **44%**	(14–79)
	**Day 29**	5/10 **50%**	(19–81)	5/8 **63%**	(24–91)	2/10 **20%**	(3–56)

**Homing IgA ASC**							
**Neg**	**Day 8**	1/9 **11%**	(0–48)	1/8 **13%**	(0–53)	0/10 **0%**	(0–31)
	**Day 29**	0/10 **0%**	(0–31)	1/9 **11%**	(0–48)	0/10 **0%**	(0–31)
**Dim**	**Day 8**	2/7 **29%**	(4–71)	2/7 **29%**	(4–71)	1/8 **13%**	(0–53)
	**Day 29**	1/10 **20%**	(0–45)	0/9 **0%**	(0–34)	0/10 **0%**	(0–31)
**High**	**Day 8**	2/5 **40%**	(5–85)	0/3 **0%**	(0–71)	1/5 **20%**	(1–72)
	**Day 29**	4/9 **44%**	(14–79)	3/7 **43%**	(10–82)	2/4 **50%**	(7–93)

a. 95% CI = Confidence interval computed via Clopper-Pearson method. Positivity defined as samples expressing gut-homing marker (ASC count > 0).							

Positive homing IgG ASC responses was more widespread, with observed responses to all three poliovirus types, the proportions of participants with α_4_β_7_^high^ IgG ASCs to poliovirus type 1 post-bOPV challenge were 50%, 62.5%, and 20% in IPV, IPV + dmLT and bOPV groups, respectively. Similarly, IgG homing ASC response rates to poliovirus type 2 post-bOPV challenge were 50%, 0%, and 100%, respectively while no participants with high levels of IgG had homing ASCs to poliovirus type 3.

### 3.4 Humoral immunogenicity

As a full polio immunization history with IPV was required for participation, the seropositivity status in the 77 per protocol participants at baseline was high; seropositivity rates were 97.4%, 93.5% and 97.4% for polio types 1, 2, and 3, respectively, and >90% in individual study groups (Table 6). Four weeks post-vaccination all participants were seropositive for all three types after IPV or IPV + dmLT vaccination. All but one participant in the bOPV group were seropositive for all three types, the exception being one person who remained seronegative for type 2. Three participants in the bOPV arm seroconverted for type 2 after vaccination, resulting in a 15% seroconversion rate, despite the absence of type 2 in bOPV. This is consistent with previously observed induction heterotypic immunity [24]. Type 1 and 2 seroconversion rates were lower for IPV + dmLT (84.0% and 92.0%) than IPV (93.1% and 100%) and type 3 seroconversion was higher after IPV + dmLT (96.0%) than IPV alone (86.2%). Geometric mean-fold increases for all three types were more than twice as high with IPV than IPV + dmLT and lower after bOPV (Table 6).

**Table 6:** Type-specific humoral neutralizing antibodies (per protocol population).

	Day 1 pre-vaccination		Day 29 post- vaccination			
Group	Geometric mean titer (95% CI)	Seropositive(%)	Geometric mean titer (95% CI)	Seropositive(%)	Geometric mean-fold rise (95% CI) ^a^	Seroconversion (%)^b^
**Poliovirus type 1**						
**IPV**	n = 30 **191** (110–329)	28/30 **93.3** (77.9–99.2)	n = 29 **18,731** (10502–33405)	29/29 **100** (88.1–100)	n = 29 **134** (53.9–334)	27/29 **93.1** (77.2–99.2)
**IPV + dmLT**	n = 27 **423** (239–748)	27/27 **100** (87.2–100)	n = 25 **25,048** (13152–47705)	25/25 **100** (86.3–100)	n = 25 **54.2** (20.3–145)	21/25 **84.0** (63.9–95.5)
**bOPV**	n = 20 **249** (129–480)	20/20 **100** (83.2–100)	n = 20 **15,657** (7770–31549)	20/20 **100** (83.2–100)	n = 20 **40.8** (13.69–122)	16/20 **80** (56.3–94.3)

**Poliovirus type 2**						
**IPV**	n = 30 **188** (93.4–378)	28/30 **93.3** (77.9–99.2)	n = 29 **45,241** (24760–82665)	29/29 **100** (88.1–100)	n = 29 **347** (122–985)	29/29 **100** (88.1–100)
**IPV + dmLT**	n = 27 **205** (100–418)	26/27 **96.3** (81.0–99.9)	n = 25 **43,251** (23053–81147)	25/25 **100** (86.3–100)	n = 25 **152** (49.5–466)	23/25 **92.0** (74.0–99.0)
**bOPV**	n = 20 **221** (96.3–508)	18/20 **90.0** (68.3–98.8)	n = 20 **241** (120–486)	19/20 **95.0** (75.1–99.9)	n = 20 **0.8** (0.22–2.7)	3/20 **15** (3.2–37.9)

**Poliovirus type 3**						
**IPV**	n = 30 **1022** (555–1882)	30/30 **93.3** (88.4–100)	n = 29 **91,419** (55229–151324)	29/29 **100** (88.1–100)	n = 29 **104** (40.7–265)	25/29 **86.2** (68.3–96.1)
**IPV + dmLT**	n = 27 **1266** (666–2406)	27/27 **100** (87.2–100)	n = 25 **52,165** (30175–90179)	25/25 **100** (86.3–100)	n = 25 **41.5** (15.1–114)	24/25 **96.0** (79.73–99.9)
**bOPV**	n = 20 **2041** (957–4355)	18/20 **90** (68.3–98.8)	n = 20 **7875** (4332–14315)	20/20 **100** (83.2–100)	n = 20 **8.6** (2.8–26.5)	10/20 **50.0** (27.2–72.8)

a. Geometric mean-fold rise and confidence interval computed via the maximum likelihood method on the difference in log_2_ titers then back-transformed.

b. ≥4-fold increase in serum neutralizing activity from baseline or post-vaccination reciprocal titer ≥ 1:8 if seronegative at baseline.

## 4 Discussion

Intramuscular addition of dmLT mucosal adjuvant did not have any meaningful impact on the safety or tolerability of IPV vaccine. There were no SAEs, deaths, or withdrawals due to an AE reported and only one related adverse event was considered to be severe – a participant in the IPV + dmLT group displayed a transient elevation of aspartate aminotransferase (AST) level which resolved spontaneously within 10 days. There were no other clinically significant changes from baseline in laboratory values, vital signs, or physical examinations. Local and systemic reactogenicity was transient and generally mild to moderate and typical of IPV vaccine in the study population of Belgian adults [25] and was only slightly increased by dmLT; the duration of local reactions was not affected by dmLT.

In adult subjects primed with IPV, four weeks after vaccination, both IPV and bOPV induced high levels of humoral neutralizing antibodies and seroconversion for all three poliovirus types (with the obvious exception of type 2 for bOPV). Neither humoral nor intestinal immunogenicity were increased by dmLT; indeed, the magnitude of humoral responses measured as geometric mean-fold rises were generally lower after IPV + dmLT than IPV alone. The addition of dmLT did not affect fecal viral shedding following bOPV challenge in comparison with IPV alone. Fecal viral shedding was generally higher in IPV-treated participants for poliovirus type 3 compared with type 1. Previous studies have hypothesized that α_4_β_7_ integrin gut homing ASCs could serve as a surrogate marker of polio vaccine-induced mucosal immune protection [26]. Further studies were recommended on subjects with and without polio vaccination exposure to generate additional data to solidify any conclusions on the relevance of these cells as such a surrogate marker, so assessment of α_4_β_7_ integrin gut homing ASCs were included in this study. There were modest levels of IgA and IgG ASC expressing the α_4_β_7_ gut homing integrin induced in response to poliovirus type 1 and few homing cells induced in response to poliovirus types 2 and 3. No differences were observed between the two IPV-treated groups in levels of fecal neutralization or fecal IgA responses, consistent with previously published findings among adults but different from infants [27], [29].

Although dmLT has been shown to have potent adjuvant capacity in preclinical animal models when administered via intramuscular or intradermal routes [11], [12], [13] and in early clinical studies [5], [14], [15] we failed to observe any impact of dmLT on the intestinal or humoral immunogenicity of co-administered IPV. Some limitations however, should be taken into account. In this study we only selected and evaluated one dose of dmLT which was generally well tolerated by the vaccinees. Further, the different age and routes of administration – adults from a high-income country given intramuscular dmLT in the present study rather than 6–59-month-old children from a LMIC who received dmLT with oral ETEC vaccine [15], as well as the high baseline seropositivity of participants in this study may have limited any measurable adjuvanting effect of the dmLT. Although the current data do not support IPV and dmLT as a solution to improving the intestinal immune response to IPV, studies of dmLT formulation in preclinical models and the tolerability of the dmLT dose used in this study may suggest the utility of another clinical assessment with higher doses of dmLT. Future evaluations should be conducted in a younger study population who are more likely to demonstrate measurable fecal neutralization and IgA responses [29]. Additionally, the interpretation of post-challenge shedding comparisons between the IPV arms and the bOPV arm were limited in this study due to ongoing vaccine shedding among 15% of bOPV recipients at the time of challenge. Future clinical assessments among OPV-naïve individuals should extend the time between vaccination and challenge to avoid this coinfection.

Alternative routes of IPV administration, e.g., intradermal injection with fractional doses of IPV (f-IPV) alone provides a viable option for dose-sparing [28], although the WHO SAGE currently recommends the use of two doses of f-IPV for routine immunization together with bOPV [30]. Individuals whose primary immunization includes IPV in conjunction with bOPV will not have the intestinal immunity required to prevent transmission of type 2 poliovirus should they be exposed [31]. In the absence of alternatives, there remains an unmet need for induction of type 2 intestinal immunity that may be addressed with development of an improved IPV or inclusion of nOPV2 which can confer intestinal immunity. Despite promising data from preclinical studies, dmLT at the dose used in the present study does not appear to address this need, and further investigation is necessary.

## 5 Funding Source

This work was funded by the Bill & Melinda Gates Foundation OPP1171111. Under the grant conditions of the Foundation, a Creative Commons Attribution 4.0 Generic License has already been assigned to the Author Accepted Manuscript version that might arise from this submission.

## Declaration of Competing Interest

The authors declare that they have no known competing financial interests or personal relationships that could have appeared to influence the work reported in this paper.

## Data Availability

Data will be made available on request.
